# Development of targeted therapies in treatment of glioblastoma

**DOI:** 10.7497/j.issn.2095-3941.2015.0020

**Published:** 2015-09

**Authors:** Yuan-Yuan Xu, Pei Gao, Ying Sun, You-Rong Duan

**Affiliations:** State Key Laboratory of Oncogenes and Related Genes, Shanghai Cancer Institute, Renji Hospital, School of Medicine, Shanghai Jiao Tong University, Shanghai 200032, China

**Keywords:** Glioblastoma (GBM), targeted therapy, blood-brain barrier (BBB), clinical trial

## Abstract

Glioblastoma (GBM) is a type of tumor that is highly lethal despite maximal therapy. Standard therapeutic approaches provide modest improvement in progression-free and overall survival, necessitating the investigation of novel therapies. Oncologic therapy has recently experienced a rapid evolution toward “targeted therapy”, with drugs directed against specific targets which play essential roles in the proliferation, survival, and invasiveness of GBM cells, including numerous molecules involved in signal transduction pathways. Inhibitors of these molecules have already entered or are undergoing clinical trials. However, significant challenges in their development remain because several preclinical and clinical studies present conflicting results. In this article, we will provide an up-to-date review of the current targeted therapies in GBM.

## Introduction

### Classification and epidemiology of glioblastoma (GBM)

Gliomas are tumors that arise from glial or precursor cells and include astrocytoma, GBM, oligodendroglioma, ependymoma, mixed glioma, malignant glioma, not otherwise specified (NOS), and a few rare histologies. According to the 2007 WHO classification of central nervous system (CNS) tumors, GBM belongs to tumors of neuroepithelial tissue and could be further subdivided into giant-cell GBM and gliosarcoma. GBM, also called grade IV astrocytoma (where I refers to the least severe and IV to the most severe), is the most common type of primary malignant brain tumor in adults, accounting for 54% of all gliomas. In the United States, the incidence rate is 3.19 per 100,000^1^^,^^2^. Approximately 0.59 to 3.69 GBM cases per 100,000 are diagnosed annually worldwide^1^^,^^3^^-^^7^. GBM is also one of the most lethal brain tumors, with only one-third of patients surviving for 1 year and less than 5% living beyond 5 years^8^^,^^9^. GBM patients survive for 12 to 15 months on average despite aggressive surgical resection and conventional therapy^10^^,^^11^.

### Morphological features of GBM

**Figure 1:**
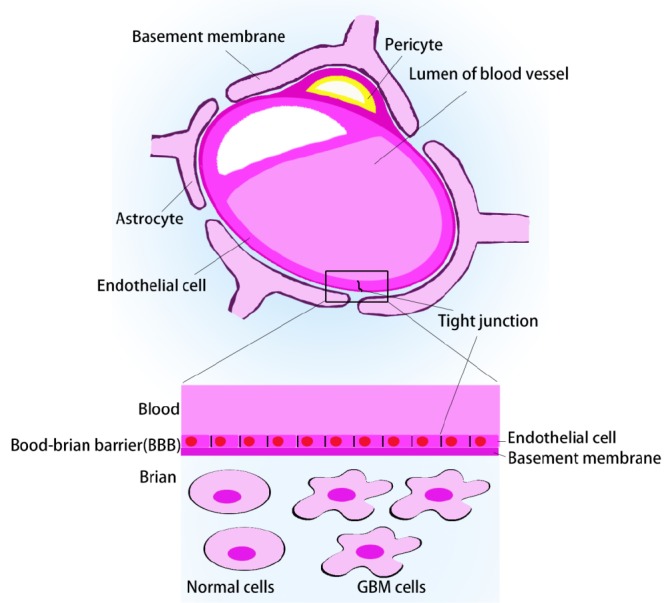
Blood-brain barrier.

Compared with other tumors, GBM possesses unique characteristics associated with its poor prognosis: (I) larger dormant glioma cells with a stronger resistance to conventional radiotherapy and chemotherapy, resulting in multi-drug resistance (MDR); (II) “crab claw-like” invasion, causing unclear borders with normal cerebral structures, preventing complete surgical resection; (III) recurrence within 2 cm of the primary tumor location rather than outside the site, making the elimination of residual glioma cells critical for radical cure and improved prognosis^12^; (IV) “Chinese chive-like” regenerative proliferation, making it possible that simple surgical excision may stimulate and further accelerate its growth rate and degree of malignancy; and (V) protection by the blood-brain barrier (BBB) (**Figure 1**) and blood-brain tumor barrier (BBTB), preventing nearly all large-molecule and 98% of small-molecule drugs from entering the CNS^13^^-^^15^. The BBTB starts to form at the later stage of glioma and resides among the brain tumor cells and microvessels^16^^-^^18^, both of which limit the penetration of conventional intravenous or oral drugs into the tumor tissue.

According to the NCCN Clinical Practice Guidelines in Oncology (NCCN Guidelines), combined chemoradiation is as a new standard of care for non-elderly patients with good performance status^2^. At present, the comprehensive model for the treatment of GBM consists of chemotherapy, surgery, and radiotherapy. The abovementioned morphological characteristics decisively imply the vision of advancing chemotherapeutic administration-targeted drug therapy.

## Targeted strategies for GBM

### Targeted therapies rationale

In targeted therapy, drugs accumulate selectively in targeted tissues, organs, cells, or intracellular structures via local drug delivery or the systemic blood circulation. These drugs could be sorted into passive and active targeting based on the delivery mechanism as follows. (I) Passive targeting: drug-loaded particles of different sizes are intercepted by different tissues because of their distinct physiological properties, such as the reticuloendothelial system (RES) in the liver and spleen or enhanced permeability and retention (EPR) effects in tumors. (II) Active targeting: drugs or carriers modified by special ligands, monoclonal antibodies, or macromolecule substances that are sensitive to certain chemicals *in vivo*, which act as “bullets”, are delivered directly and accumulate in target regions. Passive target-oriented microspheres could evade macrophage uptake and further reach the specific target sites after the modification of ligands, monoclonal antibodies, or other substances.

Considering the specific sites and features of GBM, a passive targeting strategy alone, such as the use or evasion of RES and the utilization of EPR, is insufficient for drug delivery to the tumor. Therefore, the following treatments emphasize active targeting or a combination of the two strategies.

### Receptor-mediated targeting

Certain receptors closely correlated with tumor growth are highly expressed in BBB, GBM cells, or associated blood vessels, but not in surrounding normal tissues. Given this phenomenon, surface-functionalized or conjugated traditional drugs or drug-loaded carriers with corresponding ligands are expected to guide these receptors to cross the BBB to improve therapeutic efficacy and reduce side effects.

#### Transferrin receptor (TfR)

The cellular uptake of iron-loaded transferrin (Tf) occurs through receptor-mediated transcytosis (RMT). TfR, referred to as TfR1 or CD71 in literature, is expressed at low levels in most human tissues but is highly expressed on the brain capillary endothelium, which forms the BBB, and in tumor tissue. Thus, TfR functions both in mediating transport across the BBB and internalization into cancer cells. Meanwhile, TfR2, another member of the TfR family, presents considerably lower affinity for Tf than TfR1 (25-fold lower), making it a less efficient target for TfR-mediated drug or gene delivery to brain or cancer cells^19^^,^^20^.

Zhang *et al*.^21^ prepared Tf-modified paclitaxel-loaded micelles (TRPM), wherein Tf modification significantly enhanced cellular uptake by primary brain microvascular endothelial cells to 2.4 fold that of unmodified samples, resulting in high drug accumulation in the brain after intravenous injection. Mice bearing intracranial U-87 MG glioma treated with TRPM exhibited the longest mean survival time (42.8 days). Ying *et al*.^22^ developed liposomes conjugated with Tf. Their group found that its transport ratio across the BBB model was significantly increased up to 24.9% and that the C6 glioma spheroid volume ratio was significantly lowered to 54.7%. The inhibitory rate to C6 glioma cells after crossing the BBB was significantly enhanced up to 64.0%, and the median survival time of tumor-bearing rats after administration (22 days) was significantly longer than those of other controls. Several other studies^23^^,^^24^ also showed that liposomes or nanoparticles (NPs) conjugated with Tfs can target endothelial and tumor cells, penetrating tumor cells to reach the core of tumor spheroids and providing the highest brain distribution. As a result, drug-loaded formulations present the best anti-proliferative activity against GBM cells and tumor spheroids. Chiu *et al*.^25^ showed that NPs conjugated to oxalate Tf, a variant of Tf, exhibited a higher degree of cellular association compared with native Tf-conjugated NPs, because oxalate can stabilize the iron atoms in Tf, thereby decreasing Tf iron release rate in the endosome^26^. Accordingly, conjugates of these Tf mutants with the diphtheria toxin possessed even greater potency in cytotoxicity experiments *in vitro* with GBM cell lines. Moreover, intratumoral injections into xenografted glioma tumors in a mouse model resulted in near-complete tumor regression within 8 days^27^. Therefore, using Tf variant-based therapeutics has a potential in systemic drug delivery applications for GBM treatment.

#### Low-density lipoprotein (LDL) receptor

Low-density lipoprotein receptor-related proteins (LRPs), which are structurally similar to the LDL receptors, belong to the LDL receptor family. LRPs are multifunctional RMT systems with multiple ligands, such as lactoferrin, melanotransferrin, and receptor-associated protein. Moreover, LRPs are overexpressed in BBB and glioma cells. Therefore, several BBB or glioma-targeting vectors which take advantage of the LRP RMT system have been reported^19^^,^^28^^-^^31^.

Angiopep-2, which is derived from the Kunitz domain of aprotinin, exhibits high LRP1 binding efficiency and brain penetration capability in both the *in vitro* model of BBB and *in situ* brain perfusion in mice; several research groups used Angiopep-2 for glioma-targeting delivery^31^^-^^37^. Jiang *et al*.^38^^,^^39^ developed NPs and carbon nanotubes functionalized with Angiopep-2, both of which displayed higher glioma localization and penetration. The most favorable antiglioma effects both *in vitro* and *in vivo* were observed after loading with drugs. Xin *et al*.^31^ prepared paclitaxel-loaded Angiopep-NPs that exhibit a significantly higher amount of endocytosis and enhanced inhibitory effects to U87 MG cells with significantly increased transport ratios across the BBB model. The group also observed enhanced accumulation of Angiopep-NPs in the glioma bed and infiltrating margin of an intracranial U87 MG glioma tumor-bearing *in vivo* model. Several other studies utilized Angiopep-2 to modify the delivery system, including NPs^40^, gold NPs^41^, electro-responsive hydrogel NPs^42^, *p*-coumaric acid^43^, or pluronic F127-conjugated superparamagnetic iron oxide NPs^44^, and all exerted similar findings without exception. A study applied Angiopep-2 to GBM stem cell (GSC) in which the vector was also overexpressed and, as expected, Angiopep-2 improved anti-GSC properties, such as enhanced stability, anti-proliferation, and antitumor sphere formation abilities^45^. Demeule *et al*.^46^ synthesized ANG4043 by chemically conjugating the anti-HER2 mAb with Angiopep-2. Expectedly, increased BBB permeability was observed compared with unconjugated anti-HER2 mAb. Given the susceptibility to proteolysis of Angiopep-2 (here termed ^L^Angiopep), Wei *et al*.^47^ designed a retro-inverso isomer of ^L^Angiopep, termed ^D^Angiopep. The latter demonstrated lower uptake efficiency in both bEnd.3 and U87 cells, suggesting lower binding affinity to LRP-1 of the ^D^peptide. Moreover, ^D^Angiopep was resistant to proteolysis in fresh rat blood serum, whereas more than 85% of ^L^Angiopep disappeared within 2 h. This result indicates that the susceptibility to proteolysis of ^L^Angiopep in BBB may further attenuate transcytosis efficiency. In consequence, *in vivo* ^D^Angiopep-modified micelles displayed high distribution in intracranial GBM. Therefore, the proteolytically stable ^D^Angiopep holds considerable potential in designing two-order brain tumor targeted delivery systems.

### GBM initiating cell (GIC) targeting

Evidently, this intrinsic resistance of GBM to current treatments is caused by a cell subpopulation with high resistance to radiation and chemotherapy. GICs or GSCs are responsible for tumor reinitiation and sustained growth, and are conceptualized as cancer’s locomotive engine^48^^-^^52^, making GSCs an attractive therapeutic target for GBM.

#### Bone morphogenetic proteins (BMP)

BMP belong to the transforming growth factor-β (TGF-β) superfamily of cytokines. This family of proteins was originally identified to induce bone and cartilage formation in ectopic skeletal sites *in vivo*. BMP ligands exert their activities by means of serine-threonine kinase receptors. The activation of the BMP pathway reduces glioma cell proliferation and renders GICs more susceptible to conventional therapy, so BMP treatment is considered to be a promising therapeutic tool against GBM^53^^,^^54^. Several studies have shown that BMPs can arrest cell cycle in GBM cells^55^ and suppress the tumorigenic capacity of GICs by inducing their differentiation to phenotypes with lower levels of stem cell markers^51^^,^^56^^,^^57^. Among all BMP ligands tested, BMP4 elicited the strongest effect, which could effectively inhibit not only GIC proliferation and self-renewal *in vitro*, but also tumor growth *in vivo*^53^. Mice that were intracranially injected with untreated glioma cells died after 3 to 4 months, but nearly all mice injected with BMP4-treated cells survived until the end of the experiment^58^. Univariate analysis showed that low BMP4 levels were correlated with high tumor grade. The Kaplan-Meier analysis indicated that patients with high BMP4 expression showed significantly better prognosis, highlighting the relevance of BMP4 as a predictor of survival^59^. Liu *et al*.^60^ reported that BMP4 can even reverse the MDR phenotype of tumor cells. BMP4 treatment has also been combined with bevacizumab (BEV) in GBM mouse models, and results showed that BMP4 exerted an independently favorable effect on GBM that was not synergistic with BEV treatment^61^. Chirasani *et al*.^57^ disclosed that endogenous neural stem cells secreted BMP-7, which acts as a paracrine suppressor of GICs. Animal experiments also showed that these cells would migrate to the borders of neoplastic foci to suppress GBM formation. Moreover, Tate *et al*.^62^ demonstrated that a BMP7 variant (BMP7v) decreased primary human GIC proliferation, angiogenesis, and stem cell marker expression while enhancing neuronal and astrocyte differentiation marker expression *in vitro* and *in vivo*. In addition, BMP7v reduced brain invasion, angiogenesis, and the associated mortality in an orthotopic glioma model. However, not all BMP levels were positively associated with a better clinical outcome of glioma patients. According to a report, BMP2 expression became significantly higher as the glioma’s grade advanced and the Karnofsky Performance Status score decreased^63^. Persano *et al*.^64^ reported that, besides being an effective prodifferentiation treatment for GBM-derived stem cells, BMP2 also sensitized GICs to temozolomide (TMZ) treatment by decreasing hypoxia-inducible factor 1 alpha (HIF1α) stability and consequently down-regulating O-6-methylguanine-DNA methyltransferase (MGMT), an HIF1α target, thereby promoting TMZ’s alkylating action.

#### CD133

The Pentaspan transmembrane glycoprotein family member CD133, also known as prominin-1 (PROM-1), is the best-validated marker of the cell subpopulation responsible for conferring stem cell properties to GBMs. CD133 as a marker of GSCs is also an attractive target for the delivery of targeting therapeutics. Shin and colleagues^65^ prepared CD133 antibody-conjugated immune liposomes that encapsulated gemcitabine for targeting GSCs. The *in vitro* cytotoxicity of gemcitabine was significantly enhanced through the endocytosis of CD133 overexpressed on GSCs. The anti-tumor effect was 15 times higher than that of free gemcitabine, thus presumably reflecting the specific targeting of the CD133 surface marker.

#### Telomere repeat-binding factor 2 (TRF2)

GSCs express high amounts of repressor element 1 silencing transcription (REST) factor, which may contribute to their resistance to standard therapies. Meanwhile, TRF2 stabilizes telomeres and REST to maintain the self-renewal of neural stem cells and tumor cells. Bai and coworkers^66^ showed that viral vector-mediated delivery of shRNAs targeting TRF2 mRNA depleted TRF2 and REST from GSCs isolated from patient specimens. As a result, GSC proliferation was reduced, and the level of proteins normally expressed by post-mitotic neurons (L1CAM and β3-tubulin) was increased. Depletion of TRF2 also sensitized GSCs to TMZ and increased the survival of mice bearing GSC xenografts. These findings reveal a role of TRF2 in the maintenance of REST-associated proliferation and the chemotherapy resistance of GSCs, suggesting that TRF2 was a potential therapeutic target for GBM.

#### miR-125b

Several studies^67^^,^^68^ have shown that miR-125b is necessary for GSC’s fission and insensitivity to chemotherapy. Chen *et al*.^69^ explored the functions and mechanisms of miR-125b action on TMZ-treated GSCs. The group found that miR-125b was up-regulated in TMZ-resistant cells, the inhibition of which caused a marked increase in TMZ-induced cytotoxicity and apoptosis, as well as a subsequent decrease in the resistance to TMZ in GSCs. Moreover, their study demonstrated that the pro-apoptotic Bcl-2 antagonist killer 1 (Bak1) was a direct target of miR-125b. In other words, miR-125b conferred TMZ resistance by targeting Bak1 expression^69^.

### Angiogenesis targeting

Angiogenesis is known as the rate-determining process for solid tumor growth, which is also one of the main features of tumor tissues. GBM is among the most angiogenic of malignancies^70^. Thus, angiogenesis has emerged as a primary target of drug development for GBM over recent decades. Tumor angiogenesis is involved in many stimulating (VEGF, EGF, PDGF, etc.) and inhibiting factors. Thus, numerous promising strategies exist for targeting GBM therapy, such as down-regulating the expression of stimulating factors.

#### Integrins

Integrins are a family of cell-cell and cell-extracellular matrix adhesion molecules that are implicated in various cellular processes (e.g., survival, proliferation, migration, invasion, and angiogenesis) and could thus support tumor development. In particular, α_v_β_3_ and α_v_β_5_ integrins are speculated to be key mediators of crosstalk between tumor cells and the brain microenvironment in GBM and are overexpressed on glioma cells and the vasculature. Therefore, targeting integrins and tumor microenvironment are considered to be a promising therapeutic strategy in GBM^71^^-^^74^.

#### Arg-Gly-Asp (RGD)

Arg-Gly-Asp (RGD) is a peptide that was is widely used for neovasculature targeting delivery because of its high binding efficiency with α_v_β_3_^74^^-^^77^. Notably, the binding affinity of the cyclic RGD peptide [c(RGDfK)] for integrin α_v_β_3_ is reported to be 1,000 times greater than that of the linear RGD peptide^75^^,^^76^. Therefore, Liu *et al*.^76^ conjugated [c(RGDfK)] to a cell-penetrating peptide R8 to develop the multifunctional peptide R8-RGD, which increased the cellular uptake of liposomes by two folds in comparison with separate R8. Liu *et al*.^76^ also displayed the effective penetration of 3D glioma spheroids and the BBB model *in vitro* and the glioma foci after systemic administration in C6 glioma-bearing mice. Similarly, Kibria *et al*.^75^ also selected R8/RGD to embellish PEGylated liposomes, and their results showed an enhanced cellular uptake and higher transfection efficiency in integrin α_v_β_3_-expressing cells in comparison with versions of the single ligand. Zhang *et al*.^21^ utilized [c(RGDfK)] to modify paclitaxel-loaded micelles to target integrins overexpressed in glioma cells, which showed significantly prolonged retention in glioma tumors and peritumoral tissue.

#### Cilengitide

Cilengitide, an RGD peptide mimetic and a selective inhibitor of α_v_β_3_ and α_v_β_5_, has been tested in phase I/II trials in GBM patients^74^. In several other phase I/II studies in patients with recurrent or newly diagnosed GBM, cilengitide alone or in combination with TMZ chemoradiotherapy was well tolerated and showed potential antitumor activity (particularly in tumors with a methylated MGMT promoter)^78^^-^^80^. Eisele *et al*.^81^ analyzed the patterns of progression on MRI in 21 newly diagnosed GBM patients in a phase II trial of cilengitide added to TMZ chemoradiotherapy. Their group found that adding cilengitide did not alter patterns of progression; that is, cilengitide may not induce a more aggressive phenotype at progression, nor provide anti-invasive activity in patients with newly diagnosed GBM. Therefore, in a recent randomized phase III trial, Stupp *et al*.^71^ assessed cilengitide combined with standard treatment in a subgroup of patients with GBM with a methylated MGMT promoter. The group found that the median overall survival (OS) was 26.3 months in the cilengitide group and 26.3 months in the control group (*P*=0.86). Given this result, the addition of cilengitide to TMZ chemoradiotherapy did not improve outcomes, and cilengitide should not be further developed as an anticancer drug.

#### Epidermal growth factor receptor (EGFR)

EGFR, also known as HER1 or ErbB1, belongs to the ErbB family of receptor tyrosine kinases (RTKs). Ligand binding by EGF leads to in the activation of the RTK/RAS/PI3K pathway, resulting in cellular proliferation, angiogenesis, and increased local tissue invasion, as well as resistance to apoptosis^82^^-^^84^. EGFR is amplified in 40% to 50% of GBMs. Gain-of-function EGFRvIII mutations (EGFR variant III) in nearly half of GBMs bear amplified EGFR. EGFRvIII arises from a genomic deletion of exons 2 to 7, which encode the ligand-binding domain of the receptor, generating constitutively active oncogenic RTKs. Moreover, the signaling mechanism of EGFRvIII cells can confer resistance to EGFR inhibitors (such as erlotinib and gefitinib) and promote poor long-term survival. Recent interest has focused on an anti-EGFRVIII vaccine (known as rindopepimut), which has already entered or is undergoing clinical trials^82^^,^^85^^-^^87^.

#### Inhibitors

Preclinical results have demonstrated the ability of tyrosine kinase inhibitors (TKIs) to inhibit tumor cell growth, angiogenesis, survival, and proliferation in several different EGFR-transfected GBM cell lines. However, these results do not appear to be clinically translatable, as response rates in GBM patients for numerous inhibitors, including gefitinib and erlotinib, are poor^82^. Qaddoumi *et al*.^88^ conducted a new phase II trial of erlotinib and local radiotherapy in children with newly diagnosed GBM (20 patients). The 2-year progression-free survival (PFS) for patients was 19%±8%, and only five patients remained alive without tumor progression. This result indicates that erlotinib did not change the poor outcome of children with GBM. Another phase II trial reported that the combination of radiation, TMZ, erlotinib, and BEV for the initial treatment of GBM appeared to improve PFS but did not reach the primary endpoint of improved OS^89^. Another phase II study of erlotinib and sorafenib for patients with progressive or recurrent GBM also did not meet its objective of a 30% increase in OS time compared with historical controls^90^. In the case of gefitinib, in one phase II evaluation in nearly 100 patients with newly diagnosed GBM^91^, the OS and PFS at 1 year post-RT with gefitinib were not significantly different compared with those of the historical control population. Thus, treatment with adjuvant gefitinib post-RT was not associated with significant improvement in OS or PFS.

#### Monoclonal antibodies

Despite the success of antibody-based therapy in the treatment of many other cancers, these results have not been replicated in GBM^82^. A phase II study in 2009 stratified patients depending on their EGFR gene amplification status, and both groups were administered with cetuximab intravenously. Cetuximab exerted little effect in both groups, and the median OS was 5 months, eliciting no significant correlation between EGFR status and response or OS^92^. Another similar phase II clinical study in 2012 found that patients with an EGFR amplification lacking EGFRvIII expression presented a significantly superior PFS and a numerical OS following treatment with cetuximab [median PFS, 3.03 *vs*. 1.63 months (*P*=0.006); median OS, 5.57 *vs*. 3.97 months (*P*=0.12)]. Within the subgroup of patients with EGFR amplification, patients with EGFRvIII-positive GBM showed worse survival [median PFS, 1.63 *vs*. 3.03 months (*P*=0.01); median OS, 3.27 *vs*. 5.57 months (*P*=0.08)], indicating that the type of EGFR mutation may determine the outcome of GBM patients treated with cetuximab^93^. In Chinese patients, a study of nimotuzumab in combination with TMZ and radiotherapy for newly diagnosed GBM showed that the survival times was similar to that observed in historical data of standard therapy; that is, no correlation between efficacy and EGFR expression was found^94^. The newest^95^ phase III trial involving nimotuzumab in the treatment of newly diagnosed adult GBM also showed that EGFR amplification is not correlated with clinical efficacy of nimotuzumab. This study, albeit negative, contained hypothesis-generating signals which support the evaluation of correlative, efficacy-predicting tumor parameters for nimotuzumab in GBM treatment.

#### Peptide vaccines

Rindopepimut (CDX-110) is a peptide vaccine composed of a 14-mer peptide spanning the EGFRvIII-specific exon junction site conjugated to the carrier protein KLH.

Results from previous trials, namely, ACTIVATE, ACT II, and ACTIII, confirmed the safety of rindopepimut with robust EGFRvIII-specific immune responses and demonstrated a statistical increase in median PFS and OS in vaccinated patients in comparison with a cohort treated with the care standard^85^^,^^96^^-^^98^. Schuster *et al*.^87^ performed a phase II clinical trial (ACT III) to confirm the results mentioned above. In their study, PFS at 5.5 months (~8.5 months from diagnosis) was 66%. Relative to study entry, median OS was 21.8 months, and 36-month OS was 26%. Anti-EGFRvIII antibody titers increased ≥4 folds in 85% of patients and increased with the duration of treatment. EGFRvIII was eliminated in 4 out of 6 (67%) tumor samples obtained after >3 months of therapy. A pivotal, double-blind, randomized, phase III trial (“ACT IV”) is underway.

#### Platelet-derived growth factor receptor (PDGFR)

The PDGFR family constitutes the subfamily III of RTK and is formed by PDGFR-α and PDGFR-β. PDGFRs are related to cell migration, proliferation, and survival processes^83^. Interestingly, both PDGFRs and their ligands are co-expressed in GBM, suggesting that stimulation of autocrine PDGFRs may contribute to their growth^99^. Amplifications of PDGFR-α have been extensively studied in GBM. These receptors are reported to be associated with a loss of p53 function and the secondary GBMs that typically develop from low-grade astrocytoma^100^^,^^101^. A gene expression-based GBM molecular classification has further linked PDGFR-α aberrations in patients to the proneural subclass. This GBM subclass was identified to be nonresponsive to standard TMZ and radiotherapy. Other studies have recently found PDGFR-α mutations in a fraction of diffuse intrinsic pontine glioma, which is a pediatric brain tumor with an extremely poor prognosis^101^.

At present, no PDGFR-targeting agent has been approved for GBM treatment. Studies *in vitro* showed that imatinib could inhibit GBM cell proliferation and induce growth arrest in the G_0_/G_1_ phase of the cell cycle^99^, or that imatinib could reach intratumoral concentrations similar to or higher than those in plasma in GBM regions where the BBB is disrupted as indicated in contrast-enhanced MRI^102^. Long-term exposure to imatinib could reduce the ability of cancer stem cell through the induction of cell differentiation in GBM cells^103^, and all of these strategies may indicate potential in clinical applications. However, previous clinical studies using imatinib mesylate (Gleevec^®^) for GBM patients showed no major inhibition of tumor growth or extension of survival^104^. Several multicenter trials also failed to show the efficacy of imatinib alone or in combination with hydroxyurea in the treatment of recurrent GBM^105^^,^^106^. The molecular mechanisms of action of imatinib in GBM cells remain poorly understood. Dong *et al*.^104^ investigated the effects of imatinib on PDGFR downstream signaling pathways, as well as on other cellular functions in human GBM cells. The research group found that imatinib significantly inhibited cell migration but not cell growth. The combination of imatinib and a MEK or PI3K inhibitor resulted in significant growth inhibition but did not inhibit cell migration beyond the inhibition achieved with imatinib treatment alone. This finding indicates that the imatinib treatment of malignant glioma does not result in significant inhibitory effects and should be used with caution.

#### VEGF/VEGFR

Hypoxia in GBM led to HIF-1α accumulation and further activation of several hypoxia-associated genes, including VEGF. The VEGF gene family includes six secreted glycoproteins [VEGF-A, VEGF-B, VEGF-C, VEGF-D, VEGF-E, and placenta growth factor (PlGF)]. Among these glycoproteins, VEGF-A typically localizes adjacent to perinecrotic regions within glioma pseudopalisades, increases with higher glioma grade, and is associated with poor outcome among patients with GBM. The VEGF receptor (VEGFR) family includes VEGFR-1 (Flt-1), VEGFR-2 (KDR), VEGFR-3, neuropilin-1 (NRP-1), and NRP-2, which exhibit different binding affinities of VEGF homologs. Among these receptors, VEGFR-1 and VEGFR-2 regulate angiogenesis and NRP function as VEGFR tyrosine kinase co-receptors. VEGF binding to VEGFRs on tumor blood vessels markedly enhances permeability and activates endothelial cell proliferation, survival, and migration. Moreover, GBM also expresses VEGFRs, which may function in an autocrine manner to promote tumor growth^107^.

#### Inhibitors sunitinib

A phase II trial^108^ examined the activity of sunitinib in 12 patients with newly diagnosed, non-resectable GBM. Results showed that sunitinib had no activity as a monotherapy, and further investigation of its efficacy in this setting is unwarranted. Another trial by Hutterer *et al*.^109^ also found that continuous daily sunitinib showed minimal anti-GBM activity and substantial toxicity when given at higher doses. These are the two latest reports about trials of sunitinib in recurrent GBM to date, and both were consistent with previous trials^110^^-^^112^ showing that sunitinib has no significant anti-tumor efficacy alone or in combination with others in newly diagnosed GBM.

#### Cediranib

A phase II study^113^ of cediranib in patients with recurrent GBM showed that cediranib monotherapy was associated with encouraging proportions of radiographic response and 6-month PFS. Gerstner *et al*.^114^ evaluated the effects of cediranib in combination with chemoradiation on tumor blood flow and survival in newly diagnosed GBM. Improved PFS and OS compared with historical controls, particularly in those with improved perfusion were observed. This finding was confirmed by further results of improved tumor oxygenation and survival in GBM patients who showed increased blood perfusion after cediranib and chemoradiation^115^. Batchelor *et al*.^116^ performed a phase III trial. However, the results of the group did not meet the primary end point of PFS prolongation with cediranib either as a monotherapy or in combination with lomustine versus lomustine in patients with recurrent GBM, even though cediranib showed evidence of clinical activity on several secondary end points, including time to deterioration in neurologic status and corticosteroid-sparing effects. Similarly, a phase I study of cediranib in combination with cilengitide in patients with recurrent GBM showed that the median PFS/OS and APF6 were not very promising, notwithstanding the concluding suggestion of a low incidence of pseudoprogression in newly diagnosed GBM patients treated with cediranib in combination with chemoradiation^117^.

#### Axitinib

Axitinib is a novel orally available VEGFR-TKI. Kratzsch *et al*.^118^ conducted a study with immunodeficient mice. The group established cell line- and patient-derived GBM xenografts, which were treated with axitinib. They verified that axitinib exhibited significant effects on GBM xenografts even with primary resistance to BEV in a so far untreated tumor. Another preclinical study^119^ showed for the first time the antiangiogenic effect and survival prolongation provided by systemic single-agent treatment with axitinib in preclinical orthotopic GBM models, including clinically relevant GSC models. In the newest phase II study of axitinib *vs*. standard care performed by Duerinck and coworkers^120^, axitinib had single-agent activity in recurrent GBM patients. The survival of the axitinib group was comparable with that of the contemporary control arm. Tumor response on MRI is accompanied by decreased uptake of tracers on ^18^F-FET PET scan. Further evaluation of axitinib for recurrent GBM is warranted.

#### Monoclonal antibody

BEV is a recombinant humanized monoclonal antibody that could selectively bind to and neutralize the activity of VEGF-A, thereby inhibiting binding to VEGFR. BEV received accelerated FDA approval for the treatment of progressive GBM based on radiographic response rates^121^. Goldlust *et al*.^121^ reported that their radiographic and survival outcomes with BEV following progression after VEGFR-TKIs are similar to the data from studies of BEV as initial salvage therapy. Prior exposure to VEGFR-TKIs may not preclude response to BEV, but sensitivity to BEV may be lower following more robust VEGFR inhibition^121^. In a prestigious report on a randomized trial of BEV for newly diagnosed GBM, first-line use of BEV did not improve OS in patients (median, 15.7 *vs*. 16.1 months, BEV *vs*. placebo). PFS was prolonged (10.7 *vs*. 7.3 months) but did not reach the prespecified improvement target^122^. Taal *et al*.^123^ reported the results of the first phase II trial (BELOB trial) in which single-agent BEV did not support a significant role in recurrent GBM, whereas the combination of lomustine and BEV may have more activity than either drug administered alone^124^. This combination warrants further investigation and is currently being investigated in randomized controlled phase III EORTC trial 26101^125^. Nevertheless, the addition of BEV to TMZ and hypo-IMRT, or the combination of BEV/Irinotecan (IRI) did not improve OS for patients with GBM^126^^-^^128^. Similarly, a phase II study showed that the addition of carboplatin and IRI to BEV does not improve anti-tumor activity compared to that achieved historically with single-agent BEV among BEV-naive, recurrent GBM patients^129^. In another single-institution phase II trial^130^, the combination of BEV, erlotinib, TMZ, and radiotherapy appeared to improve PFS but did not reach the primary endpoint of improved OS. These data suggest that chemosynergy with BEV may be insufficient to enhance the benefit of BEV in recurrent GBM.

### Biomarker targeting

The National Institutes of Health defines biomarkers as “characteristics that are objectively measured and evaluated as indicators of normal biologic processes, pathogenic processes, or pharmacologic responses to a therapeutic intervention”. Biomarkers have the potential to play significant roles in the diagnosis of tumor subtypes, as well as in the identification of therapeutic targets of GBM^131^. A marker can consist of alterations of the genome, epigenome, or transcriptome, proteome, and aberrant microRNAs (miRNAs). Genes that are most closely associated with GBM, such as EGFR, VEGFR, and PDGFR, are previously introduced.

Herein, we emphasize the miRNAs involved in the initiation and progression of GBM. MiRNAs are a class of short non-coding RNA sequences (18 to 24 nucleotides) that repress gene expression by interacting with the 3' untranslated regions of mRNAs^132^. MiRNAs are predicted to target more than 50% of human protein-coding genes, enabling them to perform numerous regulated roles in physiological and developmental processes^133^. MiRNA-targeted therapy is still in the initial stage, and clinical trials are under recruitment or currently running. However, several miRNAs have been selected as promising tumor biomarkers, with increased potential to reduce disease progression in combination with conventional first-line therapy for GBMs.

#### MiR-21

Altered miRNA expression in GBMs was first reported in 2005. Chan *et al*.^134^ showed that miR-21 was highly up-regulated and exhibited anti-apoptotic capabilities in GBM cell lines. Subsequently, several reports confirmed miR-21 up-regulation in GBMs. MiR-21 functions as an oncogene in the pathogenesis of GBM, and its expression is correlated with glioma grade^135^^-^^137^. In addition, miR-21 and its target genes mediate radiation resistance of GBM cells^138^^-^^140^. Therefore, miR-21-targeted therapy is a promising alternative for GBM. Ren *et al*.^141^ showed that miR-21 inhibitors in combination with 5-FU increased glioma cell apoptosis and decreased cancer cell migration. Qian *et al*.^142^ co-delivered doxorubicin and miR-21 inhibitor (miR-21i) into glioma cells, which surprisingly exhibited an anti-proliferative efficiency. A new study demonstrated that decreased tumor cell proliferation and tumor size, as well as enhanced apoptosis activation and, to a lesser extent, improvement of animal survival, were also observed in GBM-bearing mice upon systemic delivery of targeted NP-formulated anti-miR-21 oligonucleotides and exposure to sunitinib^143^.

#### MiR-181

MiR-181 family contains a, b, c, and d isoforms. The down-regulated hsa-miR-181a and hsa-miR-181b of hsa-miR-181 family were also involved in glioma oncogenesis^144^. MiR-181d was also down-regulated in human glioma samples and may act as a glioma suppressor by targeting K-ras and Bcl-2Jeny^145^. MiR-181b and miR-181d were predictive biomarkers for TMZ response, with the former possibly enhancing TMZ sensitivity via MEK1 down-regulation and the latter partly by post-transcriptional regulation of MGMT. Therefore, a combination of miR-181b or miR-181d with TMZ may be an effective therapeutic strategy for gliomas^146^^,^^147^. Further studies on miR-181 are still necessary to demonstrate a therapeutic benefit in a clinical context toward GBM targeting treatment.

## Conclusion and future perspectives

As chemotherapy for GBM has provided only a modest benefit in clinical outcome, the need for strategies with improved efficacy has prompted the development of current targeted therapies. However, drug delivery to the brain is hindered by the presence of the BBB. RMT is one of the transport systems used for nutrient transport to the brain for its healthy function. Thus, if appropriately targeted, RMT systems could help clinicians shuttle therapeutics into the brain in a noninvasive manner. At present, the most well-developed receptors known to undergo RMT are probably LfR and LRP. NPs or liposomes are always used as carriers to deliver corresponding ligands, antibodies, or peptide vaccines. In either case, the conjugated cargo gains access to the brain interstitium by “piggybacking” on the natural RMT system^19^. Notably, both LfR and LRP are overexpressed in the BBB, as well as in GBM cells, enabling the two to traverse the BBB and reach the secondary target. Therefore, achieving sequential targeting is feasible by endowing those RMTs, only expressed on either BBB or GBM cells, with an additional targeting moiety, such as RGD. The first targeting agent would allow RMT across the BBB, and the second agent would discriminate the site of action within the CNS^19^. Though the first agent was either LfR or LRP, we could also add another targeting moiety to improve penetration into the BBB or tumors, such as ACP, RGD, *p*-aminophenyl-α-D-mannopyranoside, and TLyP-1^148^. We could also change the structures of ligands to obtain their variant or isomer with better performance via physicochemical methods, and further accomplish a higher degree of cellular association or increased distribution in intracranial GBM, such as [c(RGDfK)], oxalate Tf, and ^D^Angiopep. To overcome the uptake in the RES of liver or spleen, we can coat NPs or liposomes with PEG.

Another important concern associated with GBM’s poor prognosis may be recurrence, which is possibly due to the failure to eradicate GICs. Targeting GICs initiates new potential clinical therapies and interventions. TGF-β signaling could be a potential target because it has been shown to act as an oncogenic factor in GBM and could enhance the self-renewal capacity of tumor-derived spheroids *in vitro*^149^. Like BMPs, members of the TGF-β superfamily that could block proliferation and increase GIC responsiveness to chemotherapy and low BMP levels are prognostic for poor survival in human glioma, and have been proposed as promising therapeutics. The quinoline derivative LY2109761 is a TGF-β receptor I kinase inhibitor that has been found to be active against GBM alone and to enhance the antitumor efficacy of radiation both *in vitro* and *in vivo*, particularly in GICs^150^. Moreover, GSCs are driven by overactive signaling pathways, such as PI3K/AKT/mTOR and RAS/RAF/MAPK. Evidence has been provided that sorafenib, a member of TKIs, exhibited a selective cytotoxic effect on GSCs that is partly dependent on the inhibition of the PI3K/Akt and MAPK pathways involved in gliomagenesis^151^^,^^152^. The most advantageous result is the emergence of stem cell-mediated delivery, which yielded promising preclinical results. A human clinical trial utilizing this approach is currently underway, considering incomplete distribution within the entirety of GBM of small molecule inhibitors or carriers like NPs. Therapeutic agents that have been delivered to GBMs by GIC carriers include therapeutic genes, oncolytic viruses, NPs, and antibodies (for readers interested in further discussions or opinions in this area, a review has recently been published^153^).

GBM is also characterized by high expression levels of proangiogenic cytokines and microvascular proliferation, highlighting the potential value of treatments targeting angiogenesis. Antiangiogenic treatment likely achieves a beneficial impact through multiple mechanisms of action. However, alternative proangiogenic signal transduction pathways are activated, leading to resistance development, even in tumors that initially respond. Identifying biomarkers or imaging parameters to predict the response and herald resistance is of high priority. Despite promising phase I/II clinical trial results, adding cilengitide to TMZ chemoradiotherapy did not improve outcomes. Similarly, many other inhibitors or monoclonal antibodies tended to exert unsuccessful results, as further clinical trials are underway in newly diagnosed or recurrent GBM. Simultaneously, interesting findings were also noted, such as patients who experienced gefitinib-associated adverse effects (rash/diarrhea) exhibit improved OS. Another interesting finding is the high endothelial c-Kit expression, which may define a subgroup of patients who will benefit from sunitinib treatment by achieving prolonged PFS. Even though gefitinib reached high concentrations in tumor tissue and efficiently dephosphorylated its target, the regulation of downstream signal transducers in the EGFR pathway seemed to be dominated by regulatory circuits that are independent of EGFR phosphorylation. Therefore, future studies are still warranted. Except for biomarkers associated with angiogenesis, recent reports support the potential of miRNAs as predictive biomarkers and therapeutic targets for GBMs, despite awaiting further studies, which may allow for appropriate patient enrichment. Interdisciplinary efforts seem to be required in studying the combination among RMT approaches, GSC inhibitors, antiangiogenic treatments, biomarker targeting therapies, and cytotoxic agents, which may ultimately prove successful in improving OS and convert the “undergoing clinical trials” to “FDA-approved” therapeutics for noninvasive drug delivery to GBM.
